# The Oral Cavity as a Window to Systemic Disease: Diagnostic Clues and Pre-treatment Dental Management for Interdisciplinary Trainees

**DOI:** 10.7759/cureus.111615

**Published:** 2026-06-27

**Authors:** Lina Sarbeland, Mohammad Yousuf Sultani, Nilab Afzalzada, Fraidoon Latifi, Samira Ali, Maryam Muradi, Abdul Hamid Elmyar, Mohammad Ibrahim Sultani, Tamanna Yousofi, Mursal Sharify, Rateba Kamran Qadiri, Nella Naziri Sarir, Humaira Sadat Sultany

**Affiliations:** 1 Medicine, Aria University, Mazar-i-Sharif, AFG; 2 Dentistry, Stomatology National and Teaching Hospital, Kabul, AFG; 3 Medicine, Khatam Al-Nabieen University, Kabul, AFG; 4 Psychiatry, Balkh University, Mazar-i-Sharif, AFG; 5 Obstetrics and Gynecology, Isteqlal Hospital, Kabul, AFG; 6 Public Health, Kabul University of Medical Sciences "Abu Ali Ibn Sina", Kabul, AFG; 7 Medicine, Ankara Yıldırım Beyazıt University, Ankara, TUR; 8 Medicine, Herat University, Herat, AFG; 9 Medicine, Cheragh Medical University, Kabul, AFG; 10 Medicine, Balkh University, Mazar-i-Sharif, AFG; 11 Obstetrics and Gynecology, Khair Khana 102-Bed Hospital, Kabul, AFG; 12 Medicine, Kabul University of Medical Sciences "Abu Ali Ibn Sina", Kabul, AFG

**Keywords:** dental clearance, infective endocarditis, interdisciplinary education, mronj, narrative review, oral manifestations, osteoradionecrosis, systemic disease

## Abstract

The oral cavity frequently harbors the earliest clinical manifestations of systemic diseases, yet a persistent knowledge gap exists between dental and medical training programs regarding oral-systemic correlations. Simultaneously, pre-treatment dental evaluation before oncologic therapy and cardiac surgery remains an underutilized step that, when omitted, can lead to osteoradionecrosis, infective endocarditis, treatment delays, and increased morbidity. This narrative review aimed to catalog oral diagnostic clues, organized by organ system, and to synthesize current pre-treatment dental protocols for oncologic and cardiac surgical patients, targeting interdisciplinary trainees in dentistry and internal medicine. A literature search was conducted across PubMed/MEDLINE, Scopus, Web of Science, and the Cochrane Library. Clinical studies, systematic reviews, guidelines, and consensus statements published between 2000 and 2025 were included. Oral lesions may precede systemic symptoms in conditions including pemphigus vulgaris, Crohn’s disease, leukemia, and Addison’s disease. The bidirectional relationship between periodontitis and diabetes mellitus is supported by evidence demonstrating increased rates of type 2 diabetes among affected individuals. Current pre-treatment dental protocols endorsed by major professional organizations recommend comprehensive oral evaluation, elimination of active infection, and defined healing intervals before radiation therapy. Integrating oral examination into systemic disease screening and embedding standardized dental evaluation into oncologic and cardiac surgical pathways may improve early diagnosis, reduce complications, and enhance patient outcomes. Interdisciplinary education and standardized medical-dental consultation templates remain important priorities.

## Introduction and background

The oral cavity as a mirror of systemic health

The oral mucosa, with its rapid cellular turnover, rich vascularity, and direct exposure to the external environment, serves as one of the most sensitive indicators of systemic pathology [1,2]. Oral mucosal changes may reflect hematologic, autoimmune, endocrine, gastrointestinal, dermatologic, nutritional, and infectious diseases, often appearing before systemic symptoms become clinically apparent [1-3]. For example, oral erosions precede cutaneous involvement in 50-70% of cases of pemphigus vulgaris, and cobblestone mucosa with deep linear ulcers may herald Crohn's disease months before gastrointestinal symptoms emerge, particularly in pediatric populations [1,4,5].

The knowledge gap between dentistry and internal medicine

Despite the diagnostic potential of the oral examination, a significant educational gap persists. A cross-sectional study of 244 dental students found that overall knowledge of oral manifestations of systemic diseases was intermediate, with particularly poor awareness of viral infections (11.5% correct for hepatitis viruses) and limited clinical application, as follows: only 11.1% of students reported having diagnosed a systemic disease based on oral findings [6]. Conversely, internal medicine residents receive minimal training in systematic oral examination, creating a bilateral blind spot that delays diagnosis and referral [1,6].

The clinical importance of pre-treatment dental evaluation

Pre-treatment dental evaluation is a critical yet frequently overlooked step in oncologic and cardiac surgical care pathways. Failure to address oral pathology before head and neck radiation therapy increases the risk of osteoradionecrosis (ORN), radiation caries, and xerostomia [7]. In cardiac surgical patients, untreated dental and periodontal infections serve as potential sources of bacteremia, increasing the risk of infective endocarditis and prosthetic valve infection [8-10]. Clinical protocols have shown that, while 91.7% of included records addressed pre-treatment assessment, significant gaps remain in standardizing survivorship pathways, personalizing care by oncologic modality, and defining explicit referral triggers [11].

Aim and scope

This narrative review serves a dual purpose - first, to provide a comprehensive, organ-system-based catalog of oral manifestations that serve as early diagnostic clues for systemic disease; and second, to synthesize current evidence-based pre-treatment dental protocols for patients undergoing chemotherapy, radiation therapy, and cardiac surgery. The target audience includes dental residents, internal medicine residents, and interdisciplinary care teams. This review is structured according to the Scale for the Assessment of Narrative Review Articles (SANRA) quality criteria.

Methods

Literature Search Strategy

A comprehensive literature search was conducted across PubMed/MEDLINE, Scopus, Web of Science, and the Cochrane Library. The following Boolean search strings were employed: combinations of terms related to oral manifestations, oral lesions, systemic disease, dental clearance, chemotherapy, radiotherapy, cardiac surgery, medication-related osteonecrosis of the jaw, xerostomia, gingival hyperplasia, mucositis, and dysgeusia.

Inclusion and Exclusion Criteria

Inclusion criteria encompassed clinical studies, systematic reviews, meta-analyses, clinical practice guidelines, consensus statements, and case series (≥5 patients) published in English between 2000 and 2025. Case reports with fewer than five patients, non-English articles without available translation, and animal studies were excluded.

## Review

Oral manifestations as early diagnostic clues for systemic disease

Hematologic Disorders

Hematologic conditions frequently manifest in the oral cavity due to the high vascularity and rapid turnover of oral mucosal tissues [1,2]. Iron deficiency, vitamin B12 deficiency, and folate deficiency anemias present with mucosal pallor, atrophic glossitis (smooth, depapillated tongue), angular cheilitis, and secondary oral candidiasis. Glossitis may be the presenting complaint that prompts initial evaluation (Table 1) [1].

**Table 1:** Oral manifestations of hematologic disorders. LDH: lactate dehydrogenase; AML-M5: acute monocytic leukemia

Condition	Key oral findings	Diagnostic significance	Recommended workup	References
Iron deficiency anemia	Mucosal pallor, atrophic glossitis, angular cheilitis, candidiasis	Glossitis may be the presenting complaint	CBC, serum ferritin, and iron studies	[1]
B12/folate deficiency	Glossitis, burning tongue, mucosal pallor	Hunter glossitis (beefy red tongue)	CBC, serum B12, folate, methylmalonic acid	[1]
Leukemia	Gingival hyperplasia, spontaneous bleeding, petechiae, ulceration	Gingival infiltration may be the first sign (especially AML-M5)	CBC with differential, peripheral smear, bone marrow biopsy	[1,2]
Thrombocytopenia	Petechiae, ecchymoses, prolonged bleeding	Unexplained oral bleeding → hematologic referral	CBC, coagulation studies	[1]
Lymphoma	Tonsillar/palatal swelling, non-healing ulcers, lymphadenopathy	Biopsy required for diagnosis	Biopsy, imaging, LDH	[2]

Leukemia: Gingival hyperplasia, spontaneous gingival bleeding, petechiae, ecchymoses, and mucosal ulceration are hallmark oral findings. Gingival infiltration by leukemic cells may be the first clinical sign, particularly in acute monocytic leukemia (AML-M5), and should prompt urgent hematologic evaluation with a complete blood count and peripheral smear [1,2].

Thrombocytopenia and coagulopathies: Petechiae, ecchymoses, and prolonged bleeding after minor dental procedures or trauma are characteristic. Unexplained oral bleeding in the absence of local pathology warrants investigation with a CBC and coagulation studies [1].

Lymphoma: Oral involvement may present as tonsillar or palatal swelling, non-healing ulcers, or cervical lymphadenopathy. Biopsy of suspicious lesions is essential for diagnosis [2].

Autoimmune and Connective Tissue Diseases

Oral signs are frequently the first manifestation of autoimmune diseases, and dentists play an important role in their early detection [3,12].

Systemic lupus erythematosus (SLE): Oral ulcers, typically painless and located on the hard palate, are included in the American College of Rheumatology/European League Against Rheumatism (ACR/EULAR) classification criteria for SLE. Additional findings include honeycomb plaques (silvery white, scarred plaques), raised keratotic plaques, erythema, purpura, and cheilitis [1,13]. Studies have reported that the most frequently associated oral manifestations include oral ulcers, hyposalivation, pigmentations, glossodynia, and secondary Sjögren syndrome [13].

Pemphigus vulgaris: Painful oral erosions, desquamative gingivitis, and a positive Nikolsky sign characterize this condition. Critically, oral lesions precede skin lesions in 50-70% of cases, making the oral cavity the primary site of initial presentation [1,12,14]. Diagnosis requires biopsy with direct immunofluorescence showing intercellular IgG and C3 deposits (Table 2) [14].

**Table 2:** Oral manifestations of autoimmune diseases. ANA: antinuclear antibody; DIF: direct immunofluorescence; ISG: International Study Group; BMZ: basement membrane zone

Disease	Key oral features	Biopsy/lab findings	Referral trigger	References
SLE	Painless palatal ulcers, honeycomb plaques, cheilitis	ANA, anti-dsDNA; biopsy shows interface mucositis	Recurrent painless oral ulcers + systemic symptoms	[1,2]
Pemphigus vulgaris	Painful erosions, desquamative gingivitis, Nikolsky sign	DIF: intercellular IgG/C3 (fishnet pattern)	Non-healing oral erosions preceding skin lesions	[1,3,4]
Mucous membrane pemphigoid	Desquamative gingivitis, vesicles, scarring	DIF: linear BMZ IgG/IgA/C3	Desquamative gingivitis + ocular involvement	[3,5]
Sjögren syndrome	Xerostomia, rampant caries, parotid enlargement	Minor salivary gland biopsy (focus score ≥ 1); anti-SSA/SSB	Unexplained xerostomia + keratoconjunctivitis sicca	[3,6]
Behçet disease	Recurrent aphthous ulcers (≥3 episodes/year)	Clinical diagnosis (ISG criteria); pathergy test	Recurrent aphthae + genital ulcers or uveitis	[3]

Mucous membrane pemphigoid: Desquamative gingivitis, vesicles, and scarring (particularly ocular) distinguish this from pemphigus vulgaris. Biopsy with direct immunofluorescence shows linear IgG, IgA, or C3 deposits along the basement membrane zone [12,15].

Sjögren syndrome: Xerostomia, rampant dental caries, bilateral parotid gland enlargement, and oral candidiasis are characteristic. Salivary gland biopsy demonstrating focal lymphocytic sialadenitis (focus score ≥1) is a diagnostic criterion [12,16].

Behçet disease: Recurrent oral aphthous ulcers (major, minor, or herpetiform) are a mandatory criterion in the International Study Group diagnostic criteria. Ulcers are typically painful, recurrent, and may affect any oral mucosal surface [12].

Granulomatosis with polyangiitis (GPA): A distinctive "strawberry-like" hyperplastic gingivitis with granular, erythematous, and friable gingival tissue is considered pathognomonic and should prompt antineutrophil cytoplasmic antibody (ANCA) testing and systemic evaluation [2].

Gastrointestinal Diseases

Oral manifestations of inflammatory bowel disease (IBD) are more common in Crohn's disease (CD) than ulcerative colitis (UC), with prevalence rates ranging from 8% to 50% in adult CD patients [5,17].

Crohn's disease: Specific oral manifestations include indurated mucosal tags, cobblestone mucosa, deep linear ulcers, mucogingivitis, and lip swelling with vertical fissures [5,18,19]. These lesions may precede gastrointestinal symptoms, particularly in pediatric patients, where oral changes were observed in 35.3% of children with CD at the time of diagnosis [5,17]. Oral lesions in CD are more prevalent in patients with proximal gastrointestinal tract and/or perianal involvement [5].

Ulcerative colitis: Non-specific manifestations such as aphthous stomatitis and angular cheilitis occur in both CD and UC, while pyostomatitis vegetans, characterized by pustular, vegetating lesions of the oral mucosa, is more specific to UC [17,18,20].

Celiac disease: Recurrent aphthous ulcers, dental enamel defects (particularly symmetric enamel hypoplasia of permanent teeth), and delayed dental eruption are recognized oral manifestations that may prompt serologic screening with tissue transglutaminase IgA antibodies [1].

Gastroesophageal reflux disease (GERD) and eating disorders: Dental erosion (perimolysis) is a hallmark finding. In GERD, erosion predominantly affects the palatal surfaces of maxillary teeth, while in bulimia nervosa, the pattern of erosion is more generalized but characteristically involves the palatal surfaces of anterior teeth. The pattern of erosion can help distinguish between these conditions [1].

Endocrine Disorders

Diabetes mellitus: The bidirectional relationship between diabetes and periodontitis is well established. Multiple cohort studies demonstrated that individuals with periodontitis had a 26% higher risk of incident diabetes (summarized relative risk {SRR}: 1.26, 95% CI: 1.12-1.41), while individuals with diabetes had a 24% higher risk of incident periodontitis (SRR: 1.24, 95% CI: 1.13-1.37) [21]. Large population-based studies confirmed these bidirectional associations across over 300,000 participants [22]. Randomized controlled trials demonstrate that periodontal therapy reduces HbA1c by approximately 0.43% [23]. The American Diabetes Association (ADA) 2026 standards of care recommend annual dental referral for all people with diabetes and coordination between medical and dental teams for glucose-lowering medication adjustment around dental procedures [24]. Additional oral findings include xerostomia, candidiasis, burning mouth, delayed wound healing, and periapical lesions. Severe or refractory periodontitis in a young patient should prompt screening for diabetes [1,25].

Addison’s disease: Diffuse melanin pigmentation of the oral mucosa, particularly the gingiva and buccal mucosa, may be the earliest clinical sign of primary adrenal insufficiency and should prompt evaluation with morning cortisol and adrenocorticotropic hormone (ACTH) stimulation testing [1].

Hyperparathyroidism: Brown tumors (giant cell lesions) of the jaw, loss of the lamina dura on dental radiographs, and a ground-glass appearance of the bone are characteristic radiographic findings [2].

Thyroid disorders: Macroglossia is associated with hypothyroidism, while accelerated dental development may occur in hyperthyroidism [2].

Acromegaly: Macroglossia, mandibular prognathism, diastema (spacing between teeth), and malocclusion result from growth hormone excess and may prompt endocrine evaluation [2].

Dermatologic Diseases With Oral Manifestations

Oral lichen planus: Wickham striae (reticular white lace-like patterns), typically on the buccal mucosa bilaterally, are the hallmark finding. The erosive form presents with painful erythematous and ulcerative lesions. Oral lichen planus may occur without skin involvement and carries a small risk of malignant transformation (approximately 1-2%) [2,26,27]. According to population-based data, the prevalence of oral lichen planus ranges from 1.5% in men to 2.3% in women [27]. Several classification systems have been proposed, and the coexistence of hyperkeratotic striation/reticulation with varying degrees of mucosal inflammation and a band-like infiltrate of mononuclear inflammatory cells is considered suggestive of the diagnosis [26].

Erythema multiforme/Stevens-Johnson syndrome: Hemorrhagic crusting of the lips and diffuse oral erosions with pseudomembrane formation are characteristic. Drug exposure history is critical for diagnosis. The oral mucosa is frequently involved, and lesions may be the predominant or sole manifestation [2,15].

Epidermolysis bullosa: Mucosal blistering, microstomia (from scarring), enamel hypoplasia, ankyloglossia, and early-onset periodontal disease complicate dental care and require specialized management [28]. There are four major classical heritable types (simplex, junctional, dystrophic, and Kindler), each with variable oral involvement. Dental management is essential to prevent the aggravation of soft-tissue damage and tooth loss [28].

Nutritional Deficiencies

Nutritional deficiencies frequently present with characteristic oral findings that may serve as early diagnostic clues. The major deficiencies and their associated oral manifestations are summarized in Table 3.

**Table 3:** Nutritional deficiencies and their oral manifestations.

Deficiency	Oral Manifestations	Clinical Pearl	References
Iron	Atrophic glossitis, angular cheilitis, mucosal pallor, dysphagia (Plummer-Vinson)	Most common cause of glossitis worldwide	[1]
Vitamin B12	Hunter glossitis (beefy red, smooth tongue), burning sensation, aphthae	Neurologic symptoms may coexist	[1]
Folate	Glossitis, aphthous ulcers, angular cheilitis	Often concurrent with B12 deficiency	[1]
Vitamin C (scurvy)	Gingival swelling, bleeding, tooth loosening, petechiae	Consider in malnourished, elderly, or restrictive-diet patients	[1,2]
Zinc	Dysgeusia, oral ulcers, angular cheilitis	Taste disturbance may be the primary complaint	[2]

Infectious Diseases

HIV/AIDS: Oral lesions are among the earliest and most important indicators of HIV infection [29]. Seven cardinal lesions strongly associated with HIV have been internationally calibrated as follows: oral candidiasis (pseudomembranous and erythematous), hairy leukoplakia, Kaposi sarcoma, linear gingival erythema, necrotizing ulcerative gingivitis, necrotizing ulcerative periodontitis, and non-Hodgkin lymphoma [29-31]. Hairy leukoplakia was the most common lesion (20.4%) in epidemiologic cohort studies, followed by pseudomembranous candidiasis (5.8%) [32]. Oral candidiasis in a young, otherwise healthy patient without identifiable risk factors should prompt HIV testing [29]. The HIV Medicine Association/Infectious Disease Society of America (HIVMA/IDSA) 2024 primary care guidance emphasizes systematic oropharyngeal examination, including assessment for oral hairy leukoplakia, candidiasis, aphthous ulcers, gingivitis, periodontal disease, and Kaposi sarcoma [30].

Syphilis: Oral manifestations vary by stage as follows: primary syphilis presents as a painless chancre (usually on the lip or tongue); secondary syphilis manifests as mucous patches (grayish-white plaques, often multicentric, affecting labial mucosa and tongue in 85% of cases); and tertiary syphilis may produce a gumma, typically as a necrotic palatal lesion [33]. An epidemiologic study found that 96% of oral syphilis cases were diagnosed in the secondary stage, with painful symptoms reported in 91.7% [33].

Tuberculosis: Oral TB typically presents as a chronic, non-healing ulcer on the tongue, often painful, with undermined edges. It is uncommon but should be considered in the differential diagnosis of persistent oral ulcers in endemic populations [2].

HPV-related conditions: Verruca vulgaris, squamous papilloma, and condyloma acuminatum may occur intraorally. High-risk HPV subtypes (particularly HPV-16) are associated with oropharyngeal squamous cell carcinoma [2].

Dental management before oncologic therapy and cardiac surgery

Pre-chemotherapy Dental Management

Rationale: Patients undergoing chemotherapy are at risk of bacteremia, sepsis, oral mucositis, candidiasis, and viral reactivation (particularly HSV) due to immunosuppression. Untreated dental pathology serves as a reservoir for infection during periods of neutropenia [34].

Recommended protocol: The Multinational Association of Supportive Care in Cancer/International Society of Oral Oncology (MASCC/ISOO) 2025 clinical practice statement provides comprehensive guidance for dental evaluation and management prior to treatment for hematologic malignancies and chimeric antigen receptor (CAR) T-cell therapy (Table 4) [7].

**Table 4:** Pre‑chemotherapy dental checklist. RCT: root canal treatment; ANC: absolute neutrophil count; Plt: platelet

Component	Action	Timing	Hematologic threshold	References
Comprehensive exam	Full oral examination + panoramic radiograph	As early as possible after cancer diagnosis	N/A	[1]
Caries treatment	Restore or extract teeth with advanced caries	≥ 2 weeks before chemo	ANC > 1,000/μL; Plt > 50,000/μL	[1,2]
Periodontal therapy	Scaling, root planing; extract hopeless teeth	≥ 2 weeks before chemo	ANC > 1,000/μL; Plt > 50,000/μL	[1,2]
Endodontic treatment	Complete RCT for restorable teeth; extract non-restorable teeth	≥ 2 weeks before chemo	ANC > 1,000/μL; Plt > 50,000/μL	[1,2]
Prosthetic adjustment	Adjust ill-fitting dentures to prevent mucosal trauma	Before chemo initiation	N/A	[1]
Patient education	Oral hygiene instruction, mucositis prevention, candidiasis awareness	Before chemo initiation	N/A	[1]
Day-of-procedure labs	CBC with differential on day of invasive dental procedure	Day of procedure	ANC > 1,000/μL; Plt > 50,000/μL	[2]

Key recommendations include the MASCC/ISOO 2025 clinical practice statement, which recommends comprehensive oral examination with panoramic and periapical radiographs prior to chemotherapy initiation, along with elimination of active infection through extractions, endodontic treatment, and periodontal therapy [7]. Patients should also receive education regarding oral hygiene and expected oral complications during treatment. Hematologic thresholds for dental procedures generally include an absolute neutrophil count above 1,000/µL and a platelet count above 50,000/µL [34]. Whenever feasible, dental treatment should be completed at least two weeks before initiation of chemotherapy. The American Academy of Family Physicians (AAFP) medical clearance guidelines confirm that patients undergoing chemotherapy without radiation may receive routine dental care, provided a CBC is performed on the day of planned treatment to verify adequate counts [34].

Special considerations: CAR T-cell therapy introduces additional considerations into the dental treatment plan before and immediately after cytoreductive therapy, as outlined in the MASCC/ISOO 2025 statement [7]. Hematopoietic stem cell transplant (HSCT) patients require particularly aggressive pre-treatment dental optimization given the prolonged immunosuppression and risk of graft-versus-host disease affecting the oral cavity [11,7].

Pre-radiation Therapy Dental Management (Head and Neck)

Rationale: Radiation therapy (RT) to the head and neck causes xerostomia and salivary gland dysfunction, dramatically increasing the risk of dental caries and its sequelae, including dentoalveolar infection and osteoradionecrosis. Radiation-related caries and dental hard-tissue changes can appear within the first three months after RT. Osteoradionecrosis (ORN) risk is highest with radiation doses > 60 Gy to the mandible [7,34].

Pre-RT dental protocol: Current multidisciplinary recommendations for pre-radiotherapy dental management include the following. Recommended measures include a comprehensive oral and head-and-neck examination, with radiographic evaluation of all teeth, and risk assessment for caries and periodontal disease based on oral hygiene, existing dental conditions, radiographic findings, and patient motivation. Teeth with poor prognosis, including those affected by moderate-to-severe periodontal disease, periapical pathology, advanced caries, or partially erupted third molars within the planned radiation field, should ideally be extracted at least two weeks before initiation of radiotherapy. Clinical studies have demonstrated an increased risk of osteoradionecrosis when extractions were performed within two weeks of oncologic treatment, compared with earlier intervention [35,36]. However, radiotherapy should not be unnecessarily delayed solely because of the timing of dental extraction, even if it is delayed, when oncologic control may be compromised [7]. Additional recommendations include fabricating custom fluoride trays, prescribing daily high-potency topical fluoride for long-term use, and educating patients on xerostomia management, oral hygiene, and candidiasis prevention.

During and post-RT management: Goals during and after RT include managing xerostomia (sialagogues such as pilocarpine or cevimeline, salivary substitutes, increased hydration), preventing trismus (jaw exercises), evaluating and treating oral candidiasis, and preventing radiation caries through continued fluoride use [37]. Dental recall visits should occur at least every six months, or more frequently for patients with xerostomia or new caries.

Osteoradionecrosis prevention and management: The ISOO-MASCC-American Society of Clinical Oncology (ASCO) 2024 guideline provides evidence-based recommendations for ORN prevention and management. Before post-radiation dental interventions, a review of the radiation therapy plan, with attention to mandibular and maxillary dose distributions, is recommended [7]. For teeth located in high-risk osteoradionecrosis (ORN) zones, conservative alternatives such as root canal therapy, crowns, or fillings should be considered unless recurrent infection or severe pain necessitates extraction [7]. Oral antibiotics before and after invasive dental procedures may be beneficial in patients at elevated ORN risk [7]. In selected cancer-free patients undergoing invasive dental procedures, pentoxifylline and tocopherol may be prescribed beginning at least one week before and continuing four weeks after the procedure [7]. Routine prophylactic hyperbaric oxygen therapy prior to dental extraction is not routinely recommended because current evidence supporting its benefit remains limited [7]. Osteoradionecrosis risk factors and evidence‑based prevention and management strategies are summarized in Table 5.

**Table 5:** ORN risk factors, prevention strategies, and management. ORN: osteoradionecrosis; RT: radiation therapy; HBO: hyperbaric oxygen

Categories	Details	References
Risk factors	Radiation dose > 60 Gy to mandible; dental extractions post-RT; poor oral hygiene; periodontal disease; tobacco/alcohol use; immunosuppression	[1,2]
Prevention - pre-RT	Extract hopeless teeth ≥ 2 weeks before RT; daily topical fluoride; oral hygiene optimization; address modifiable risk factors	[1,3,4]
Prevention - post-RT	Avoid extractions in irradiated field when possible; use alternatives (RCT, restorations); antibiotics peri-procedurally; pentoxifylline + tocopherol peri-procedurally	[1]
Management - conservative	Chlorhexidine rinses (0.12% or 0.2%), antibiotics, pain control, removal of loose sequestra	[1]
Management - surgical	Debridement or resection for advanced (stage 3) disease or failed conservative therapy	[1]
Not recommended	Routine prophylactic HBO before dental extractions	[1]

Pre-cardiac Surgery Dental Management

Rationale: Oral infections, particularly periodontitis and apical periodontitis, serve as potential sources of bacteremia that may lead to infective endocarditis (IE) or prosthetic valve infection following cardiac surgery [8-10]. The RAND/University of California, Los Angeles (UCLA) consensus report established that a consensually validated protocol for dental screening of patients awaiting cardiac interventions is considered mandatory by professionals with expertise in dental, cardiology, and cardiac surgery [9,38].

Dental screening protocol: Recommended components of dental screening include a comprehensive dental and periodontal examination, panoramic radiography to identify periapical pathology, periodontal bone loss, and retained roots, and the elimination of active dental and periodontal infections [8,9,39]. Timing considerations should be individualized according to surgical urgency; elective procedures should ideally be completed before surgery, whereas urgent or emergent procedures may require abbreviated or deferred dental screening [9]. Tailored preventive counseling is also recommended to reduce the long-term risk of infective endocarditis associated with dental pathology [39].

Infective endocarditis prophylaxis: Current recommendations emphasize the critical role of good oral health and regular access to dental care for all patients [40]. No changes to the 2007 viridans group streptococci infective endocarditis (VGS IE) prevention guidelines were recommended, with continued emphasis on the critical role of good oral health and regular access to dental care for all patients [40]. The 2020 ACC/AHA Valvular Heart Disease guideline provides a Class IIa recommendation for antibiotic prophylaxis prior to dental procedures involving manipulation of gingival tissue, the periapical region, or perforation of the oral mucosa in patients with prosthetic cardiac valves, prosthetic material used for cardiac valve repair, a history of infective endocarditis, unrepaired cyanotic congenital heart disease, repaired congenital heart disease with residual shunts or valvular regurgitation at or adjacent to a prosthetic patch or prosthetic device, and cardiac transplant recipients with valvular regurgitation due to a structurally abnormal valve [41].

The preferred prophylactic regimen is a single 2-g oral dose of amoxicillin, given 30-60 min before the procedure. For patients with penicillin allergy, cephalexin, doxycycline, or azithromycin may be used. Notably, clindamycin is no longer recommended for antibiotic prophylaxis before dental procedures [42].

Anticoagulation management during dental procedures: Management of antithrombotic therapy around dental procedures is a frequent source of clinical uncertainty. Current evidence supports the vitamin K antagonist (VKA) approach. Multiple randomized trials comparing VKA continuation versus interruption for minor dental procedures (single- and multiple-tooth extractions, endodontic procedures) showed no significant increase in bleeding with VKA continuation [43]. The American College of Chest Physicians (ACCP) 2022 guideline supports continuing VKAs (with INR: ≤ 3.5) for most dental procedures, with local hemostatic measures (tranexamic acid rinse, sutures, topical hemostatic agents) as needed [43].

Direct oral anticoagulants (DOACs): For procedures with minimal bleeding risk (e.g., minor dental procedures), DOACs may be continued; if there is concern about excessive bleeding, they may be discontinued on the day of the procedure [44]. Heparin bridging is not required for perioperative DOAC management [44].

Antiplatelet agents: Aspirin and clopidogrel should generally be continued for simple dental procedures. Elective dental care should be deferred for six weeks after bare-metal stent (BMS) placement and six months after drug-eluting stent (DES) placement to avoid premature discontinuation of dual antiplatelet therapy [34]. Cardiac conditions requiring infective endocarditis prophylaxis and considerations for anticoagulation during dental procedures are summarized in Table 6.

**Table 6:** Cardiac conditions requiring IE prophylaxis and anticoagulation management for dental procedures. CHD: congenital heart disease; VKA: vitamin K antagonists; DOAC: direct oral anticoagulants; IE: infective endocarditis; BMS: bare-metal stent; DES: drug-eluting stent

Categories	Details	References
IE prophylaxis - high-risk conditions	Prosthetic valves (mechanical, bioprosthetic, transcatheter); prior IE; unrepaired cyanotic CHD; repaired CHD with residual defects; cardiac transplant with valvulopathy	[8-10]
IE prophylaxis - regimen	Amoxicillin 2 g PO 30-60 min before procedure; penicillin allergy: cephalexin, doxycycline, or azithromycin; clindamycin no longer recommended	[8,10]
VKA (warfarin)	Continue for dental procedures if INR ≤ 3.5; use local hemostatic measures	[38]
DOACs	May continue for minor dental procedures; discontinue day-of if bleeding concern	[39]
Antiplatelet agents	Continue aspirin/clopidogrel for simple procedures; defer elective dental care 6 weeks post-BMS, 6 months post-DES	[40]

Medication-Related Osteonecrosis of the Jaw (MRONJ)

Implicated medications: MRONJ is a debilitating side effect of antiresorptive and antiangiogenic agents that can lead to progressive bone destruction in the maxillofacial region [45]. The current position and clinical practice guideline identify the following implicated medications: bisphosphonates, denosumab, antiangiogenic agents, and emerging targeted therapies [46,47]. Intravenous bisphosphonates such as zoledronic acid and pamidronate carry a substantially higher risk of MRONJ than oral formulations, including alendronate and risedronate, with the risk increasing with duration of therapy [47]. Denosumab, a receptor activator of nuclear factor kappa B ligand (RANKL) inhibitor, demonstrates an MRONJ risk comparable to that of intravenous bisphosphonates in oncology-dose settings [47]. Antiangiogenic agents, including bevacizumab and sunitinib, particularly when combined with antiresorptive therapy, have also been associated with the development of MRONJ [48]. Emerging agents, such as mTOR inhibitors (e.g., everolimus and temsirolimus) and immunomodulators, have also been linked to MRONJ, although evidence remains limited [47].

Risk stratification: Risk stratification is essential for clinical decision-making in patients receiving antiresorptive therapy [46,47]. Oncology-dose antiresorptives, such as zoledronic acid 4 mg intravenously monthly and denosumab 120 mg subcutaneously monthly, are associated with the highest MRONJ risk, with reported incidence ranging from 1% to 15% depending on treatment duration and concomitant medications. In contrast, osteoporosis-dose antiresorptives, including alendronate 70 mg orally weekly and denosumab 60 mg subcutaneously every six months, are associated with substantially lower MRONJ incidence rates of approximately 0.001-0.01%. Additional risk factors include dental extractions, concomitant corticosteroid use, poor oral hygiene, periodontal disease, ill-fitting dentures, diabetes, smoking, and chemotherapy [47].

Prevention strategies: Current expert recommendations support a comprehensive pre-treatment dental evaluation before initiating antiresorptive or antiangiogenic therapy, including elimination of active dental disease and optimization of oral hygiene [46-48]. Patient education regarding oral hygiene maintenance is strongly encouraged. Invasive dental procedures, including extractions and implant placement, should be avoided during therapy whenever possible; when extraction is unavoidable, conservative surgical technique with primary wound closure is recommended. Drug-holiday strategies remain controversial. The current expert recommendation notes that, in selected patients receiving osteoporosis-dose oral bisphosphonates for more than four years, or more than four years with concomitant systemic risk factors, temporary discontinuation for approximately two months before and three months after invasive dental procedures may be considered in consultation with the prescribing physician, although supporting evidence remains limited [47].

Diagnosis and staging: MRONJ is defined as exposed bone or bone that can be probed through an intraoral or extraoral fistula in the maxillofacial region, persisting for more than eight weeks, in a patient with current or previous treatment with antiresorptive or antiangiogenic agents, and with no history of radiation therapy to the jaws [47]. Established staging approaches include the American Association of Oral and Maxillofacial Surgeons (AAOMS) staging system, Common Terminology Criteria for Adverse Events (CTCAE) 5.0, or the International Task Force on Osteonecrosis of the Jaw (ONJ) staging system [46].

One commonly used staging system classifies MRONJ into several clinical stages [47]. Patients considered "at risk" have no apparent necrotic bone despite treatment with antiresorptive or antiangiogenic agents. Stage 0 includes non-specific clinical findings, radiographic abnormalities, and symptoms without exposed necrotic bone. Stage 1 is characterized by exposed or necrotic bone or by fistula probing to bone in asymptomatic patients without evidence of infection. Stage 2 includes exposed or necrotic bone associated with pain, erythema, purulent drainage, or clinical evidence of infection. Stage 3 disease involves exposed or necrotic bone, with pain and infection, and may be accompanied by complications such as pathologic fracture, extraoral fistula, oroantral or oronasal communication, or osteolysis extending to the inferior border of the mandible or the sinus floor [47].

Management: Available evidence remains insufficient to determine the best intervention for managing MRONJ, though several approaches have shown promise [49]. Current management strategies for MRONJ vary according to disease stage and symptom severity [47,49]. Conservative management for stages 0-2 includes chlorhexidine rinses, systemic antibiotics such as amoxicillin/clavulanate or clindamycin in penicillin-allergic patients, pain control, and removal of loose sequestra [47]. Surgical management is generally reserved for refractory stage 2 disease and stage 3 disease and may involve debridement, sequestrectomy, or segmental resection [47]. Emerging therapies, including pentoxifylline and alpha-tocopherol (PENT-E), teriparatide, photobiomodulation, photodynamic therapy, and platelet-rich plasma, have shown potential to enhance healing outcomes, although additional validation through larger controlled studies remains necessary [45].

Interdisciplinary framework and clinical implications

The Role of the Internist in Dental Clearance

What the medical consultation should include: Effective medical-dental communication requires that consultations provide specific and actionable clinical information rather than a vague statement indicating that the patient is "cleared for dental treatment" [34]. Important components include current medical conditions and their management status, a complete medication list with particular attention to antiresorptive agents, anticoagulants, antiplatelet agents, and immunosuppressants, as well as relevant laboratory values such as INR for patients receiving vitamin K antagonists, complete blood count with differential for oncology patients, glomerular filtration rate for renal dosing considerations, and HbA1c for diabetes management. Consultations should additionally summarize oncologic history, including radiation fields and doses, chemotherapy agents and timing, immunotherapy status, and provide specific recommendations regarding procedural timing, antibiotic prophylaxis, and medication management.

Common pitfalls in medical-dental communication: Several recurring communication failures have been identified in medical-dental coordination [34]. Common pitfalls include non-specific clearance statements that fail to address procedural risks, omission of bisphosphonate or radiation therapy history, inadequate communication of hematologic parameters in oncology patients, and a lack of standardized consultation templates across institutions.

Proposed standardized medical-dental consultation template: A standardized template should include the following fields: (1) patient demographics and referring dentist contact, (2) planned dental procedure(s), (3) active medical diagnoses, (4) complete medication list with start dates, (5) relevant laboratory values with dates, (6) oncologic history (radiation fields/doses, chemotherapy agents/dates), (7) specific procedural recommendations (antibiotic prophylaxis, anticoagulation management, hematologic thresholds), and (8) follow-up plan and emergency contact.

The Role of the Dentist in Systemic Disease Detection

Oral examination as a screening tool: A systematic oral examination should follow a standardized sequence as follows: lips → buccal mucosa → tongue (dorsal, ventral, lateral) → floor of mouth → hard and soft palate → gingiva → teeth → temporomandibular joint [1,2]. Red flags requiring medical referral include unexplained oral ulcers persisting more than two weeks, unexplained oral pigmentation, spontaneous gingival bleeding without local cause, unexplained xerostomia, and suspicious masses or lymphadenopathy [1,2].

When to refer to internal medicine: Dentists should consider referral to internal medicine in several clinical scenarios. These include unexplained recurrent oral ulcers that may suggest conditions such as systemic lupus erythematosus, Behçet disease, inflammatory bowel disease, or celiac disease, diffuse oral pigmentation without medication-related or racial explanation that may indicate Addison’s disease; spontaneous gingival bleeding or gingival hyperplasia without local causes, raising concern for leukemia or thrombocytopenia; severe or refractory periodontitis in young patients that may warrant diabetes screening; unexplained xerostomia associated with rampant caries suggestive of Sjögren syndrome; and strawberry-like gingivitis, which may indicate granulomatosis with polyangiitis.

Integrating oral health screening into primary care: Current evidence remains insufficient to assess the balance of benefits and harms of screening and preventive interventions for oral health conditions in adults by primary care clinicians (I statement) [50]. However, population-based data suggest that dental caries affects more than 90% of US adults, approximately 42% of adults older than 30 years have periodontal disease, and significant barriers exist to providing oral health services in primary care settings, including variable clinician access and familiarity with interventions [50]. Despite the insufficient evidence rating, the United States Preventive Services Task Force (USPSTF) noted that potential screening approaches (oral clinical assessments, standardized risk assessment instruments) are non-invasive and are unlikely to cause serious harm [50].

Drug‑Induced Oral Conditions: A Shared Responsibility

Drug‑induced oral conditions are common and represent a shared responsibility between prescribing physicians and dental providers. The major categories and representative examples are outlined below and summarized in Table 7.

**Table 7:** Common drug-induced oral conditions. ACE: angiotensin-converting enzyme; mTOR: mechanistic target of rapamycin; CCB: calcium channel blocker; RT: radiation therapy; HSCT: hematopoietic stem cell transplant; NSAID: nonsteroidal anti-inflammatory drug; MRONJ: medication-related osteonecrosis of the jaw

Oral conditions	Commonly implicated drug classes	Key clinical features	Prevention/management	References
Xerostomia	Anticholinergics, antidepressants, antihistamines, diuretics, opioids	Dry mouth, ropey saliva, glossy tongue, rampant caries	Sialagogues, oral lubricants, fluoride, hydration; minimize dose	[8,9]
Gingival hyperplasia	CCBs (nifedipine, amlodipine), cyclosporine, phenytoin	Enlarged, fibrotic gingiva, especially interdental papillae	Oral hygiene, drug substitution, gingivectomy if persistent	[8,10]
Oral mucositis	5-FU, methotrexate, doxorubicin, mTOR inhibitors, RT	Erythema, ulceration, pseudomembranes, pain	Cryotherapy, palifermin (for HSCT), basic oral care, pain management	[38]
Dysgeusia	ACE inhibitors, metronidazole, lithium, chemotherapy	Altered taste, metallic taste	Usually reversible with drug discontinuation	[8]
Hyperpigmentation	Minocycline, antimalarials (hydroxychloroquine), amiodarone, oral contraceptives	Discoloration of oral/perioral tissues	Limit duration; usually reversible; laser/cryotherapy if persistent	[8]
Angioedema	ACE inhibitors, NSAIDs	Lip/tongue swelling	Antihistamines, corticosteroids; drug avoidance	[8]
Oral candidiasis	Inhaled corticosteroids, broad-spectrum antibiotics	White coating on tongue that wipes off	Rinse mouth after inhaler use; antifungal treatment	[8]
MRONJ	Bisphosphonates (IV > oral), denosumab, antiangiogenic agents	Exposed necrotic bone, pain, infection	Pre-treatment dental optimization as discussed in MRONJ subhead	[8,39]

Xerostomia is a common adverse effect of more than 500 medications that list xerostomia as a side effect. Major drug classes include anticholinergics, antidepressants (tricyclics, selective serotonin reuptake inhibitors {SSRIs}), antihistamines, antihypertensives, diuretics, opioids, bronchodilators, anxiolytics, and skeletal muscle relaxants [48,51]. Management includes encouraging water intake, sialagogues (pilocarpine 5 mg TID or cevimeline 30 mg TID), oral lubricants, fluoride supplementation, and avoidance of caries-promoting habits [48,51].

Drug-induced gingival hyperplasia has been associated with several medication classes, including calcium channel blockers (particularly nifedipine and amlodipine), cyclosporine, and anticonvulsants (phenytoin and carbamazepine) [16,48]. Management includes prescribing the lowest effective dose, promoting meticulous oral hygiene, considering drug substitution, and surgical excision (gingivectomy) if not reversed after three to six months of drug discontinuation [48].

Oral mucositis is commonly associated with chemotherapeutic agents (particularly 5-fluorouracil, methotrexate, and doxorubicin), targeted therapies (such as mTOR inhibitors and everolimus), and radiation therapy. Mucositis affects up to 40% of patients receiving standard-dose chemotherapy and up to 80% of patients receiving high-dose chemotherapy or head and neck radiation (Table 8) [52].

**Table 8:** Oral manifestations of gastrointestinal diseases. UC: ulcerative colitis; GERD: gastroesophageal reflux disease; GPA: granulomatosis with polyangiitis; ANCA: antineutrophil cytoplasmic antibody

Disease	Oral findings	Specificity	Diagnostic clue	References
Crohn's disease	Cobblestone mucosa, deep linear ulcers, mucosal tags, lip swelling with fissures	Highly specific	May precede GI symptoms, especially in children	[1-3]
Ulcerative colitis	Aphthous stomatitis, angular cheilitis, pyostomatitis vegetans	Pyostomatitis vegetans more specific to UC	Pustular vegetating oral lesions → colonoscopy	[4-6]
Celiac disease	Recurrent aphthae, enamel defects, delayed eruption	Moderate	Symmetric enamel hypoplasia → tTG-IgA screening	[52]
GERD	Dental erosion (palatal surfaces of maxillary teeth)	Low (pattern-dependent)	Erosion pattern distinguishes from bulimia	[52]
Bulimia nervosa	Perimolysis, parotid enlargement, mucosal erythema	Moderate	Palatal erosion of anterior teeth + parotid swelling	[52]
GPA	Strawberry gingivitis (granular, hyperplastic)	c-ANCA/PR3; biopsy shows granulomatous vasculitis	Hyperplastic gingivitis + respiratory/renal symptoms	[52]

Dysgeusia has been linked to medications such as ACE inhibitors, metronidazole, lithium, chemotherapy agents, and antibiotics, such as clarithromycin, metronidazole, are common causes. Taste disturbance is usually reversible upon drug discontinuation [48]. Medication-related osteonecrosis of the jaw is primarily associated with bisphosphonates, denosumab, and antiangiogenic agents, as discussed in the MRONJ subhead [47,48].

Discussion

Summary of key findings: This review demonstrates that the oral cavity is a rich diagnostic environment in which systemic diseases frequently manifest before other clinical signs become apparent. Hematologic malignancies, autoimmune diseases (particularly pemphigus vulgaris and SLE), inflammatory bowel disease, and endocrine disorders all have well-characterized oral presentations that, when recognized, can accelerate diagnosis and improve outcomes. Simultaneously, pre-treatment dental evaluation before oncologic therapy and cardiac surgery is supported by robust guideline evidence from the National Comprehensive Cancer Network (NCCN), ISOO-MASCC-ASCO, American Heart Association (AHA), and AAOMS, yet remains inconsistently implemented across institutions [7,9,35,40,47].

Educational implications for residency training: The knowledge gap identified in dental student surveys underscores the need for integrated oral-systemic curricula in both dental and medical residency programs [6]. Proposed educational interventions include incorporating systematic oral examination training into internal medicine residency curricula, integrating modules on recognition of systemic disease into dental residency programs, and developing standardized clinical decision-support tools for oral-systemic referral pathways.

Limitations of the current evidence: Several limitations should be acknowledged. First, much of the evidence on oral manifestations as diagnostic clues derives from case series and cross-sectional studies, with limited prospective data on the diagnostic yield of systematic oral screening. Second, pre-treatment dental protocols vary across institutions and countries, and the optimal timing and extent of dental intervention before oncologic therapy remain areas of active investigation. Third, the USPSTF 2023 I statement highlights the insufficient evidence base for oral health screening in primary care settings [50]. Fourth, as a narrative review, this work is subject to selection bias in the literature included.

Future directions: Several areas warrant further investigation, including prospective studies evaluating the diagnostic yield of systematic oral examination in primary care and specialty settings, standardization of pre-treatment dental protocols across oncologic and cardiac surgical centers, and development of personalized survivorship pathways according to treatment modality [11]. Additional priorities include development and validation of AI-assisted oral lesion detection tools for point-of-care use, randomized controlled trials evaluating standardized medical-dental consultation templates and their impact on patient outcomes, and further research into MRONJ prevention strategies, including the role of drug holidays and emerging medical therapies [12].

## Conclusions

The oral cavity serves as a valuable diagnostic window into systemic disease, with oral manifestations frequently preceding systemic symptoms in conditions ranging from autoimmune diseases to hematologic malignancies. Pre-treatment dental evaluation is a critical yet underutilized step in oncologic and cardiac surgical care pathways, supported by current multidisciplinary recommendations. Interdisciplinary collaboration between dentists and internists, facilitated by standardized communication templates, integrated educational curricula, and bidirectional referral pathways, has the potential to improve early diagnosis, reduce treatment complications, and enhance patient outcomes. The persistent knowledge gap between dental and medical training programs represents both a challenge and an opportunity for educational innovation.
